# Impact of Factors Associated with Promoting Family Functioning in Fathers on Children with Neurodevelopmental Disorders

**DOI:** 10.31662/jmaj.2025-0055

**Published:** 2025-11-28

**Authors:** Shiori Ishida, Tomone Takahashi

**Affiliations:** 1 School of Health Sciences, Faculty of Medicine, Shinshu University, Matsumoto, Japan; 2 School of Humanities and Social Sciences, Faculty of Education, Shinshu University, Nagano, Japan

**Keywords:** children with neurodevelopmental disorders, fathers’ childcare, impact on the family, family functioning

## Abstract

**Introduction:**

Raising a child with a neurodevelopmental disorder can present significant challenges for families and is a known risk factor for increased parental stress. Successfully addressing these challenges by promoting family functioning is considered crucial, with particular recent emphasis on optimal paternal involvement. Interventions targeting fathers can help foster stronger family relationships, reduce stress, and enhance overall family functioning, which in turn supports the growth and neurodevelopment of the child. The present study examined the impact of fathers’ successful childcare behaviors and attitudes on the family, specifically focusing on children with neurodevelopmental disorders.

**Methods:**

An online survey was conducted with couples who had children aged 2 to 12 years diagnosed with or suspected of having a neurodevelopmental disorder. The survey explored the characteristics of fathers considered successful in parenting, assessing their effect on the child’s neurodevelopment and the partner’s parenting stress. Structural models were then constructed to quantify these relationships.

**Results:**

The survey results indicated that fathers who effectively managed childcare responsibilities indirectly contributed to reducing the child’s behavioral problems by significantly alleviating the spouse’s parenting stress.

**Conclusions:**

Fathers may play an important role in indirectly mitigating behavioral issues in children with neurodevelopmental disorders. Fostering self-awareness in fathers and helping them build positive relationships with their partners appear to be essential components of effective parenting. These aspects should be incorporated into father training programs to better support family dynamics and, by extension, the growth of children with neurodevelopmental disorders.

## Introduction

Early childhood is a period of significant growth, making effective parental involvement crucial for child development ^(1), (2)^. Children with neurodevelopmental disorders such as autism spectrum disorder (ASD), attention deficit hyperactivity disorder (ADHD), and specific learning disorder often face daily challenges, even without intellectual delays, which may require specialized support ^(3), (4)^. Parenting children with these disorders is more demanding than raising neurotypical children, with parents reporting higher levels of stress and poorer mental health ^(5), (6), (7)^. These conditions can lead to ineffective parenting, thereby impairing the child’s development and causing anxiety, aggression, delayed communication, and diminished social skills ^(8), (9), (10), (11)^. Supporting both parents’ mental and physical health is therefore essential for optimal care. Earlier studies have emphasized the importance of fathers in maintaining positive relationships within the family and society to support children with neurodevelopmental disorders ^(12), (13)^. However, understanding of fathers’ roles and relationship challenges remains limited, and formal support systems for fathers are still underdeveloped ^(14)^.

### Family functions required for a successful parenting system

Several family functions are considered essential for the growth of children with neuro

developmental disorders, including economic status, emotional support, socialization, education, and protection ^(15), (16), (17), (18)^. Enhancing these factors requires effective cooperation between parents. For example, mothers are expected to provide emotional support, manage childcare, promote communication, and collaborate with fathers to establish a cooperative childcare system with defined roles ^(19), (20), (21), (22)^, while fathers are expected to actively participate in childcare, spend quality time with children, empathize with the children’s emotions, maintain open communication, and support family cooperation ^(23), (24)^. Although changes in parental perceptions, family relationships, and fathers’ roles are emerging as factors that reduce parenting stress, few studies have directly examined their impact on parenting stress reduction and child development.

### Factors affecting family functioning

Family functioning is influenced by individual factors within the family. Earlier studies on parents of children with ASD revealed that low self-efficacy was associated with negative emotions towards parenting and diminished confidence in parenting abilities ^(25)^. These findings indicated that better self-understanding could increase parenting confidence and help overcome the challenges of raising children with neurodevelopmental disorders. Although other general parental abilities for effective child-rearing have been identified, such as hardiness and stress coping, little is known about the extent to which fathers possess those traits and how they impact parenting ^(26), (27)^.

### Need for participation of fathers in family functioning

The positive influence of paternal involvement on children and families has been widely acknowledged, with evidence highlighting its importance in childcare for promoting the child’s mental development and its contribution to family functioning ^(28)^. Jellett et al. ^(29)^ suggested that reducing parenting stress in fathers could increase marital relationship satisfaction and represent a key factor in coping with the challenges of raising a child with ASD. In such families, however, fathers are often less involved in childcare, resulting in high levels of maternal stress from the burden of child-raising. Increasing the time fathers spend in childcare, with enthusiasm and a proactive approach, can help alleviate maternal stress and improve intervention patterns for children with ASD ^(30)^. Thus, it is critical to identify the factors related to fathers’ childcare behaviors and attitudes that contribute to family functioning, along with their precise impact on the family.

### The current study

This study builds on prior research showing that certain parental factors negatively impact children with developmental disorders ^(8), (9), (10), (11)^. Rempel et al. ^(31)^ found that supporting fathers in building positive relationships with their children led to increased knowledge, improved attitudes, and stronger affection from parents, fostering early attachment and supporting infant development. In line with these findings, our study explored the role of fathers in promoting the growth of children with neurodevelopmental disorders by targeting parents of children with diagnosed neurodevelopmental disorders or receiving developmental support for suspected ASD, ADHD, or other conditions. We specifically examined the paternal factors related to family functioning and their effects on children’s behavioral issues and maternal stress. This study hypothesized that factors such as stress coping, hardiness, self-awareness, and positive relationships would improve children’s behavior and reduce spousal stress. To test this hypothesis, we conducted a survey on fathers’ personality traits and relationships with the child and spouse, using paired responses from both parents.

## Materials and Methods

### Participants and procedures

An online survey targeting parents of children aged 2 to 12 years with a diagnosis of neurodevelopmental disorders (excluding intellectual disability or specific learning disorder alone) or receiving developmental support services due to suspected ASD or ADHD was conducted from October 1, 2023, to May 30, 2024. Participants were asked to respond only after providing consent, with both parents required to answer all relevant questions. Respondents were recruited mainly from families living in rural areas or small- to medium-sized cities and were uniformly Japanese. Informed consent was obtained by an opt-in method through the distribution of an explanatory letter (including the study’s significance, purpose, methods, and expected results) and a consent form, which was voluntarily completed by each participant.

### Measures

The survey items were primarily based on a questionnaire developed in a prior study ^(32)^ to identify factors that enhanced family functioning and parenting. Using these factors along with published literature, we constructed a model to examine the characteristics of fathers who were considered successful in child-rearing, as well as their impact on the growth of children with neurodevelopmental disorders and the reduction of parenting stress in the spouse.

### Basic information

Participants were asked to provide such demographic information as age,

educational background, employment status, and age of the child.

### Characteristics of successful fathers

The observed variables constituting the latent variable in our analysis consisted of eight items. In addition to the items of stress coping and hardiness already deemed essential for parenting, six items were selected to evaluate the impact of fathers who were successfully caring for children with neurodevelopmental disorders.

1) Stress Coping: The Stress Coping Scale ^(33)^ was used to measure fathers’ tendency to choose strategies for coping with stress in various situations, not limited to child-rearing. Higher scores indicated a greater ability to cope with stressors. The total score (range: 0-42) was used in the analysis.

2) Hardiness: The Hardiness Scale ^(34)^ was adopted to measure hardiness, an individual characteristic that reflects resilience to stress. Negative items were reverse-scored, and higher scores indicated greater hardiness. The total score (range: 15-75) was used in the analysis.

3) Self-Understanding: Recently reported self-understanding items ^(32)^ were designed to measure fathers’ understanding of themselves. Higher scores suggested a greater level of self-understanding. The total score (range: 5-45) was used in the analysis.

4) Father’s Role Behavior and Awareness, and 5) Parenting Stress Index (PSI) (Short Version) ^(35)^ - Parental Aspect: To assess fathers’ role behavior and awareness of their role in parenting, we employed fathers’ role behavior and awareness items ^(32)^ as well as the PSI Parental Aspect. For the fathers’ role behavior and awareness, higher scores indicated a greater level of paternal role awareness and behavior. The total score (range: 8-40) was used in the analysis.<S> </S>Regarding the PSI Parental Aspect, positive items were reverse-scored, with lower scores indicating lower parenting stress. The total score (range: 10-50) was used in the analysis.

6) Relationship with the Child and 7) PSI (Short Version) Child Aspect ^(35)^: Items related to the father-child relationship ^(32)^ and the PSI Child Aspect ^(35)^ were adopted to assess fathers’ relationships with their children and their parenting-related stress. For the relationship with the child, the total score (range: 8-40) was used to evaluate the quality of the father-child relationship, with higher scores indicating a better relationship. Regarding the PSI Child Aspect, lower scores indicated lower parenting stress. The total score (range: 9-45) was used in the analysis.

8) Spousal Relationship: Spousal relationship items ^(32)^ were used to assess the quality of the relationship between the father and spouse. Negative items were reverse-scored, with higher scores indicating a better spousal relationship. The total score (range: 16-80) was used in the analysis.

### Children’s development

Child development was assessed by both parents using the Japanese version of the Strengths and Difficulties Questionnaire (SDQ) ^(36)^, which consisted of 25 items across five subscales: emotional symptoms (ES), conduct problems (CPs), hyperactivity/inattention (HI), peer problems (PPs), and prosocial behavior (PB). The SDQ subscales measuring difficulties (ES, CP, HI, and PP) and strengths (PB) were summed, with higher scores on difficulty subscales indicating more severe issues. Examples of items included “Often seems worried or anxious” for ES, “Often fights with other children or bullies them” for CP, “Cannot sit still for long” for HI, “Gets along better with adults than with other children” for PP, and “Considers the feelings of others” for PB. Responses were recorded on a 4-point Likert scale from “Does not apply” to “Completely applies.” Positive items were reverse-scored, and higher scores indicated greater difficulty for the child. The total score (range: 0-50) was used in the analysis.

### Maternal parenting stress

The parenting stress of the spouse was assessed using the PSI Child and Parent Aspects. Responses were recorded on a 5-point Likert scale from “Strongly disagree” to “Strongly agree.” Positive items were reverse-scored, and higher total scores indicated increased levels of parenting stress. The total score (range: 19-95) was used in the analysis.

### Data analysis

A model was constructed using structural equation modeling (SEM) with the latent variables of the above-mentioned characteristics of successful fathers, children’s development, and maternal parenting stress. Briefly, path coefficients between the three latent variables yielded factor loadings ranging from −1.00 to +1.00. For positive relationships, factor loadings were generally interpreted as <0.40: low association with the factor, 0.40-0.69: moderate association, and ≥0.70: high association. Strong factor loadings were considered to indicate that the variable explained the factor well. SEM was performed using IBM SPSS Amos 27 Graphics (IBM Corporation, Armonk, NY).

Descriptive statistics were used to present an overview of the participants’ basic characteristics. The characteristics of successful fathers were based on the eight indicators described above (Stress coping, Hardiness, Self-Understanding, Fathers’ Role Awareness and Behavior, PSI Parental Aspect, Relationship with the Child, PSI Child Aspect, and Spousal Relationship). Children’s development was assessed by two indicators, the fathers’ and mothers’ evaluations, for each of the five SDQ subscales (ES, CP, HI, PP, and PB). Maternal parenting stress was measured by two indicators based on the parenting and child-related aspects of the PSI. The validity of children’s development was ensured by defining it through both fathers’ and mothers’ evaluations. A total of five models were constructed, each treating one of the five SDQ subscales as a latent variable, to evaluate the relationships among father factors, children’s development, and maternal parenting stress, as well as to provide quantitative insights into their specific impact. Our data analysis aimed to examine the relationships among latent variables, measure the latent variables, evaluate model fit, and analyze direct and indirect effects. Model fit was calculated using the Comparative Fit Index (CFI) and the root mean square error of approximation (RMSEA). For all analyses, a p < 0.05 was considered significant.

## Results

### Summary of participants

The study sample consisted of 53 couples (i.e., 53 fathers and 53 mothers) who provided consent for participation in the online survey. The mean age of fathers and mothers was 42.7 ± 7.2 years and 40.8 ± 5.9 years, respectively. Table 1 summarizes the demographic characteristics (age, education, and employment status) of the participants, along with the age of children receiving support for neurodevelopmental disorders with or without a formal diagnosis.

**Table 1.** Respondent details.

Variable		Fathers (n=53)		Mothers (n=53)	
		Mean ± SD	(range)	Mean ± SD	(range)
Age	(years)	42.7 ± 7.2	(27-58)	40.8 ± 5.9	(27-54)
		n	(%)	n	(%)
Decade of life	20’s	1	(1.9)	2	(3.8)
	30’s	20	(37.7)	19	(35.8)
	40’s	24	(45.3)	28	(52.8)
	50’s	8	(15.1)	4	(7.5)
Educational background	High school graduate	12	(22.6)	10	(18.8)
	Graduation from vocational school or junior college	12	(22.6)	18	(34.0)
	Graduation from university or higher	29	(54.8)	25	(47.2)
Employment situation	Full-time job	52	(98.1)	6	(11.3)
	Part-time job	1	(1.9)	17	(32.1)
	No job	0		30	(56.6)
Age of child with developmental disorder or receiving support	<5 years	4	(7.5)		
	5-10 years	36	(67.9)		
	>10 years	13	(24.6)		

SD, standard deviation

### Paternal factors significantly related to successful fathers

Factor loading of the eight selected variables describing the characteristics of successful fathers in childcare as the latent variable showed significant (all p < 0.05) and were mostly strong associations (≥0.70) with the SDQ subscales of problem (CP, HI, PP, and ES) and positive (PB) behavior items in children with developmental disorders (Table 2). Notably, the factors of Self-Understanding, Fathers’ Role Awareness and Behavior, Relationship with the Child, and Spousal Relationship displayed factor loadings of .80 or higher (all p < 0.001).

**Table 2.** Influence of the 8 factors describing father’s success in parenting on SDQ subscale items (factor loading) and on maternal parenting stress (path coefficient).

	CP		HI		PP		PB		ES	
(Factor loading)										
Stress coping	.82	***	.85	***	.75	***	.78	***	.71	**
Hardiness	.85	***	.87	***	.78	***	.81	***	.75	**
Self-understanding	.94	***	.95	***	.91	***	.92	***	.89	***
Father’s role	.90	***	.92	***	.86	***	.88	***	.84	***
PSI - Parental aspect	.58	***	.62	***	.49	***	.52	***	.45	***
Relationship with the child	.92	***	.93	***	.87	***	.89	***	.85	***
PSI - Child aspect	.70	***	.75	***	.62	**	.65	***	.58	*
Spousal relationship	.93	***	.94	***	.90	***	.91	***	.88	***
(Path coefficient)										
Maternal parenting stress	-.91	***	-.93	***	-.86	***	-.89	***	-.82	***

* *p* <.05, ** *p* <.01, *** *p* <.001 PSI, parenting stress index

### Impact of successful father factors on children’s development and maternal parenting stress

**Figure 1.**
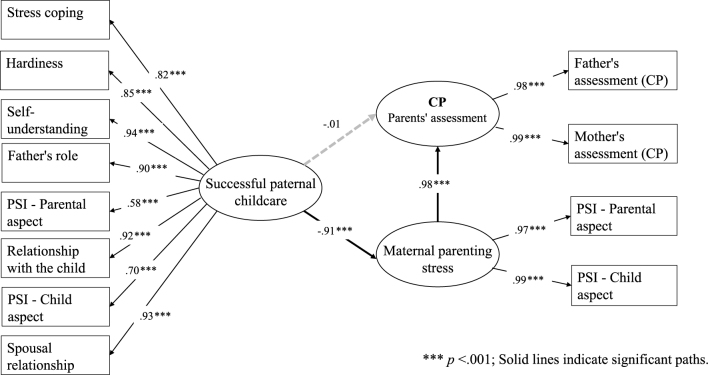
SEM results of successful paternal childcare and maternal parenting stress with CP as the dependent variable. *** p <.001; Solid lines indicate significant paths. CP: conduct problem; SEM: structural equation modeling.

**Figure 2.**
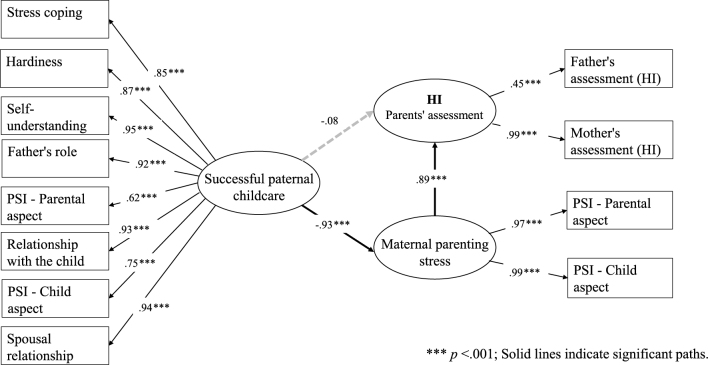
SEM results of successful paternal childcare and maternal parenting stress with HI as the dependent variable. *** p <.001; Solid lines indicate significant paths. HI: hyperactivity/inattention; SEM: structural equation modeling.

Using SEM, we examined how successful paternal involvement in childcare influenced the behavioral characteristics of children with neurodevelopmental disorders as well as maternal parenting stress. Among the five SDQ subscales, no significant direct paths were detected between successful paternal childcare and improved children’s behavioral problems. However, CP and HI findings revealed that successful paternal childcare significantly and strongly reduced maternal parenting stress, which in turn significantly and strongly improved children’s behavioral problems (Figure 1 and 2). In the remaining three subscales (ES, PP, and PB), the paths linking paternal factors and maternal stress were all statistically significant, but those between maternal stress and children’s behavioral characteristics were not.

Regarding CP, the path coefficient between paternal factors and maternal stress was significant at −0.91 (p < 0.001) and between maternal stress and children’s CP was significant at 0.98 (p < 0.001), although the direct path between paternal factors and children’s CP was not (Figure 1). Concerning HI, the path coefficient between paternal factors and maternal stress was significant at −0.93 (p < 0.001), and between maternal stress and children’s HI was significant at 0.89 (p < 0.001) (Figure 2). The model fit with paternal factors and maternal stress was limited for both CP (CFI = 0.837, RMSEA = 0.215) and HI (CFI = 0.764, RMSEA = 0.203).

## Discussion

The present study revealed that fathers who successfully engage in parenting significantly reduce their spouse’s parenting stress and, by extension, may improve the behavioral characteristics of their child with neurodevelopmental disorders. This result aligns with the findings of Kamiyama and Noro ^(37)^. Cheng et al. ^(38)^ and Nicolaus et al. ^(39)^ also showed that positive relationships between couples and between fathers and children could reduce maternal parenting stress, although they did not address the direct influence of fathers on children. The data from this study provide new insights by quantitatively demonstrating that successful father involvement may at least indirectly reduce children’s behavioral problems through the attenuation of spousal stress, as observed for CP and HI. Opondo et al. ^(40)^ earlier used the SDQ as an indicator of children’s problem behaviors and showed improvements in the total SDQ score. The current study was able to specify those findings into more specific behavioral domains.

Particularly high factor loadings were observed for the paternal scores related to self-understanding, relationships with the spouse and children, and the father’s role in parenting, thereby highlighting these factors in successful parenting. Our findings corroborate previous research demonstrating that parental self-understanding enhances relationships with children and spouses and increases motivation for parenting ^(25), (41), (42)^. However, while earlier studies focused mainly on mothers or both parents, the present investigation was able to provide results directly related to fathers’ self-understanding, role, and relationships with the child and spouse.

From our findings, it can be inferred that successful fathers play an important role in at least indirectly reducing the problem behaviors of children with neurodevelopmental disorders, establishing them as key figures in supporting child growth. Specifically, fostering self-understanding and positive relationships with the spouse and child appear to be crucial elements for fathers to successfully take on parenting responsibilities.

### Fathers as the behind-the-scenes actors to reduce problem behavior and promote growth in children with neurodevelopmental disorders

Although our findings could not confirm that fathers directly reduced the problem behaviors of children with neurodevelopmental disorders, the results suggested that fathers might indirectly contribute to this by easing the spouse’s parenting stress. In Japan, mothers primarily handle child-rearing, leaving fathers with fewer opportunities to engage with their children. This may limit their exposure to problem behaviors and awareness of their influence on the child’s development.

The reduction of maternal parenting stress from successful paternal involvement with the child was significantly related to a decrease in the problem behaviors of CP and HI. This observation suggests that by stabilizing the father’s well-being, both the child and the spouse, who experiences more direct parenting stress, may also benefit. Garcia-Lopez et al. ^(43)^ reported that fathers’ stress significantly impacted mothers’ relationship satisfaction. When mothers were satisfied with their marital relationship, the effect extended beyond them, alleviating stress for both parents and enhancing the mother’s ability to cope with the challenges related to raising a child with ASD. It can thus be inferred that building a positive marital relationship leads to better outcomes for the child. These findings are consistent with other research showing that positive marital relationships contribute to family well-being, happiness, cohesion, and cooperation ^(44), (45)^. Overall, better supporting fathers will not only strengthen their parenting attitudes and behaviors but also serve as emotional support for the spouse and promote the child’s development. This underscores the importance of interventions focused on fathers. Specifically, fathers of children with neurodevelopmental disabilities need safe spaces to express their issues and concerns. Individual counseling services and clear information about their child’s behavior can support self-understanding and reduce stress. Moreover, peer interactions may help ease isolation and promote engagement with other fathers. Flexible programs, such as short evening or weekend workshops, may address fathers’ needs while accommodating their working schedules and family commitments. Lastly, encouraging joint participation with spouses and discussing role-sharing from the father’s perspective can enhance mutual understanding and ultimately improve the caregiving environment.

### Interventions for fathers to enhance family functioning

Our findings emphasize the importance of fathers recognizing their role in child-rearing, improving self-awareness, and fostering positive family relationships. This supports existing evidence that self-understanding enhances emotional stability and promotes growth for both parents and children ^(41), (42)^. Helping fathers identify personality traits and weaknesses is considered essential. One possible intervention toward this aim is a father-focused program that encourages self-awareness and strengthens relationships with the spouse to boost both parents’ confidence in child-rearing. Programs such as those investigated by Rempel et al. ^(31)^ have improved fathers’ attitudes, increased affection towards infants, and enhanced paternal involvement, thereby contributing to child development. Group-based programs addressing the unique challenges of raising children with neurodevelopmental disorders have also shown benefits by reducing children’s behavioral problems and improving parent-child interactions ^(46)^. These initiatives also serve as physical places where fathers can share their concerns, which may reduce feelings of isolation and motivate positive involvement. Designing support flexibly in response to fathers’ needs, suggesting concrete roles within the household, and providing opportunities for small successes may create momentum and promote more active paternal engagement in family functioning.

### Study limitations

This study has several limitations, including a small sample size, inclusion of severe cases, possible recall bias, and variability in children’s symptoms that may have impacted maternal stress. Furthermore, the diagnoses of ASD and ADHD were based on parent reports, with details on the diagnostician or method being unavailable in some cases. Given that diagnoses may be made by non-specialists in Japan, including child psychiatrists, pediatricians, and physicians in community support centers, this study focused more on observed traits and support needs than diagnostic precision. Nonetheless, inconsistent diagnostic procedures may have affected sample selection, and future studies should employ physician-issued diagnoses and standardized tools on ASD, ADHD, and any possible co-occurrence. In addition, parental mental health was assessed at a superficial level in this investigation and was not treated as a key variable due to its sensitive nature and lack of objective measures. We are currently planning to incorporate more detailed assessments under appropriate ethical protocols. Furthermore, the involvement of child guidance centers and welfare programs, while not explicitly excluded, was not systematically assessed but might have influenced caregiving or child development. Future research should account for this aspect as well. Similarly, the broader social context should be analyzed in greater depth to clarify how environmental factors, such as maternal and paternal grandparents, affect both children and caregivers. Lastly, as larger-scale studies become feasible, stratified analyses by developmental stage should be conducted to identify more specific needs for parenting support. As the SEM model in this study showed limited fit, larger and more diverse samples are expected to refine the model and clarify the paternal influences on parenting stress and child behavior, thereby contributing to better family functioning.

### Conclusions

This study showed that fathers exhibiting successful paternal characteristics in childcare may contribute to reducing children’s problem behaviors through a reduction in maternal parenting stress. Based on these results, revisiting approaches to father-focused parenting support will help empower fathers, raise their confidence in parenting, strengthen family functioning, and ultimately promote the growth of children with neurodevelopmental disorders.

## Article Information

### Acknowledgments

The authors would like to express their deepest gratitude to the experts who contributed to this study, as well as to the participants who completed surveys. The authors would also like to thank Trevor Ralph for his English editorial assistance.

### Author Contributions

Conceptualization, Methodology, Formal analysis, Investigation, Writing-Original draft preparation, Project administration, and Funding acquisition: Shiori Ishida. Investigation, Writing-Reviewing, and Editing: Tomone Takahashi.

### Conflicts of Interest

Each author signed a form for disclosure of potential conflicts of interest. No authors reported any financial or other conflicts of interest in relation to the work described. The authors declare that they have no known competing financial interests or personal relationships that could have appeared to influence the work reported in this paper.

### Approval by the Institutional Review Board

Approved by the Ethics Committee of Shinshu University School of Medicine (no. 5578) and was conducted in accordance with the Declaration of Helsinki.
